# Improving performance intelligence for governing an integrated health and social care delivery network: a case study on the Amsterdam Noord district

**DOI:** 10.1186/s12913-021-06558-2

**Published:** 2021-05-28

**Authors:** Véronique L. L. C. Bos, Niek S. Klazinga, Dionne S. Kringos

**Affiliations:** Department of Public and Occupational Health, Amsterdam UMC, Amsterdam Public Health Research Institute, University of Amsterdam, Meibergdreef 9, 1105 AZ Amsterdam, the Netherlands

**Keywords:** Performance intelligence, Performance measurement, Integrated care, Governance, Health services, The Netherlands, Triple aim, Amsterdam Noord, Network, Population management

## Abstract

**Background:**

A guiding principle of a successful integrated health and social care delivery network is to establish a governance approach based on learning, grounded in a data and knowledge infrastructure. The ‘Krijtmolen Alliantie’ is a network of health and social care providers with the ambition to realize such a performance intelligence driven governance model in line with the Triple Aim. This study seeks to identify what performance intelligence is available and how it can be improved.

**Methods:**

This case study was conducted in the district of Amsterdam Noord, the Netherlands, and employed 23 semi-structured interviews with stakeholders in health and social care, a feasibility analysis of available administrative data, and a reflection meeting with board members of the ‘Krijtmolen Alliantie’. Information needs for performance intelligence by the stakeholders were mapped and a data landscape of the district covered by the network was drafted. Finally, in the reflection meeting with board members of the ‘Krijtmolen Alliantie’ the information needs and data landscape were aligned with governing needs, resulting in priority domains around which to strengthen the data infrastructure for governance of the integrated health and social care delivery network.

**Results:**

The ‘Krijtmolen Alliantie’ encompasses a network of providers with a diverse range of catchment areas. There are indicators on population health and welfare, however they have limited actionability for providers due to a misalignment with their respective catchment areas. There is a barrier in data exchange between health and social care providers. It is difficult to construct one indicator for per capita cost in the Dutch health data infrastructure as health and social care are subdivided in financing siloes. Priority domains for improvement of performance intelligence for the ‘Krijtmolen Alliantie’ are: 1) Per capita and per patient cost data integration that would allow combined accountability through aligning financial incentives to facilitate integrated care, and 2) combined patient experience and outcome measures to reflect network quality of care and patient experience performance.

**Conclusion:**

Available performance intelligence lacks actionability for the governance of integrated care networks. Our recommendation is to align performance intelligence with the regional governance responsibilities of stakeholders for health and social care delivery.

**Supplementary Information:**

The online version contains supplementary material available at 10.1186/s12913-021-06558-2.

## Background

The delivery of health and social care in integrated care networks has become a necessity to provide people centred care [1]. Concepts like Population Management and the Triple Aim, redirect measuring performance of the delivery of health and social care from single performance domains by siloed organizations, towards measuring the performance of integrated care networks [2, 3]. Performance intelligence is an essential tool to govern integrated delivery systems, to redefine value of health and social care provision, and monitor outcomes [4–8]. Performance intelligence is defined as “the structured approach to acting on health policies, using knowledge and information generated by the application of scientific methods to comparable healthcare data to systematically measure indicators of health systems performance” [9]. The actionability, defined as fitness for purpose and use, of performance intelligence is important in order for it to contribute to governance [10]. However, knowledge on how to create actionable performance intelligence to guide governance of integrated health and social care networks is still limited [11, 12]. There is no ‘off the shelf’ framework available to measure performance of integrated care delivery [13], and lessons on hindering and facilitating factors to achieve improved outcomes in national and local level integrated care approaches illustrate the complexity worldwide [14–18]. An awareness of the particularities of the local setting when implementing integrated care [19], and including perceptions of stakeholders involved in coordinating care delivery across organizations, is known to be important [20]. In the Netherlands, healthcare reforms in 2007 and 2015 decentralized social care to the level of the municipalities, and ‘de Juiste Zorg op de Juiste Plek’ (the right care at the right place) launched by the Ministry of Health, Welfare and Sport in 2018 is one of the strategies to organise health and social care needs of the patient closer to home [21]. The ‘Krijtmolen Alliantie’ (KMA) is a network of health and social care providers in the Amsterdam Noord district in the Netherlands who have been cooperating for about 10 years with the goal to realize a better experience of care for patients, improved health outcomes for the population, and lower per capita costs in line with the Triple Aim proposed by the Institute for Healthcare Improvement [2]. The alliance can be best described as a network of linked separate existing organizations, addressing common concerns with the aim to refer patients to the right care at the right time. Initial project based focus of the alliance has been on specific patients groups and their related care pathways. Information is exchanged on a ‘provide when asked and ask when needed basis’ and quality and financial accountability is still organization based. However, the alliance has been moving towards a more coordinated network with a focus on the population as a whole by pooling some finances of its members to organize multidisciplinary case managers for vulnerable groups and introduce monitoring of the flow of emergency room and hospital visits to identify vulnerable groups who need special attention in the district. Recently the KMA has decided to strengthen their cooperation through enhancing their shared performance intelligence.

This paper provides a case study on how to prioritize domains to strengthen performance intelligence in integrated care networks. The objective of this research is to investigate the perceived need for performance intelligence among the members and stakeholders of an integrated care network, explore possibilities for generating performance intelligence given the current data landscape, and identify priority domains for improvement of performance intelligence to govern the integrated care network. To do so we address the following questions: 1) Who are the members of the KMA and their stakeholders in the health and social care district Amsterdam Noord, and what are their roles and information needs? 2) What data is currently collected by health and social care providers and their stakeholders in the district covered by the network, which exchanges of information already take place and what performance intelligence is available to support the Triple Aim goals? And, 3) What priority domains to improve performance intelligence can be identified in Amsterdam Noord?

## Methods

### Research setting

The KMA serves the Amsterdam Noord district, one of the 8 districts in Amsterdam. The district is geographically separated from the rest of the city of Amsterdam by the IJ river and has a population of 97.200 inhabitants [22]. High social care needs in combination with an already existing alliance of health and social care providers makes Amsterdam Noord an interesting case study for districts and regions that wish to establish performance intelligence to support further integration of health and social care governance. In Table 1 a brief description of the research setting is provided.

**Table 1:** Key characteristics of the Amsterdam Noord district [22]

Characteristics		Amsterdam Noord	Benchmarks^**a**^		
			AMS (including Noord)	AMS (excl. Noord)	Netherlands
**Inhabitants (2019)**	Total	97.200	862.987		
	65+ years of age	15%	13%		19%
	Under 20 years of age	23%	19%		22%
**Geography (2019)**	Neighbourhoods	15	99		
	Area (ha)	6382.85	16,473.34		
	Postal codes	1020–1039			
**Social economic status**	Average income per household per year (x€1.000; 2017)	34.5	39.0		
	Unemployment rate (%) in labour force (2018)	6	5		
	Percentage low education level (15–74 year olds; 2017)	34	24		
**Health provision (2019)**	Hospitals	1			
	General practitioner practices	27			
	Pharmacies	15			
**Health and social care usage 65+ years of age (Gemeentezorgspiegel, 2018)**	Long-term Care Act (WLZ) average cost per inhabitant	€3.532		€2.999	€3.532
	Social Support Act (WMO) average cost per inhabitant	€963		€815	
	Healthcare Insurance Act (ZVW) average cost per inhabitant	€6.690		€6.133	€5.732

^a^Benchmarks given: *N* Amsterdam Noord district, *NL* Netherlands, *AMS* city of Amsterdam, *AMS-N* city of Amsterdam excluding Amsterdam Noord district

### Data collection

A mixed method study was conducted using semi-structured interviews (*n* = 23) with KMA members and stakeholders of the Amsterdam Noord district, a feasibility analysis exploring available administrative data, and a reflection meeting with the governing board of the KMA. The data was collected from November 2019 to May 2020. For the semi-structured interviews (*n* = 23) we used stratified purposive sampling [23]. The interviewees needed to represent a) KMA members (*n* = 11), and b) key stakeholders in health and social care in the Amsterdam Noord district (*n* = 12) (citizens/patients, healthcare financiers, and other health and social care providers). The interviewee list was validated with the chair of the KMA (see Additional file 1 for the validated interviewee lists). Interviewees were invited via the KMA secretariat and were informed, and asked to consent to contribute to the content of the research and the processing of the interview data. The interviews were held in Dutch, approximately 30 min per interview, audio recorded, transcribed, and shared with the interviewees to check for accuracy. The interview guide was structured in line with the Triple Aim vision of the network for the district and contained questions on the provider organizations and their role in Amsterdam Noord, what data is collected, what information is currently used to govern services delivery, if there are any initiatives for exchanging data or integrated governance, and what is the current need for performance intelligence (for the translated interview guide see Additional file 2). The database used for the feasibility analysis was the ‘Gemeentezorgspiegel’ database in collaboration with Vektis (the healthcare information centre that functions as the national claims data centre for health care consumption reimbursed via the Dutch Health Insurance Act) and the municipality of Amsterdam. The database consists of claims data from health insurance companies and public health authorities representing yearly about 75.3 billion euro (about 96% the Dutch national health budget in 2018) [24]. The data consists of reimbursements within the Long-term Care Act (WLZ); regulating care for citizens with a permanent 24 h care need, the Social Support Act (WMO); regulating municipal responsibility for supplementing citizens in their care need, and the Healthcare insurance Act (ZVW); regulating the right to medical specialist care and health insurance for all citizens. The reflection meeting with the board members of the KMA (*n* = 7) was organized digitally, as alternative to an in-person meeting, due to COVID-19 restrictions, in the presence of three researchers, one to present the findings, one to moderate the discussion, and one to take notes of the discussion.

### Analysis

To answer research question 1, pieces of transcript were grouped that relate to the role of the interviewed organization and their catchment areas in a table. Then all pieces of transcript containing content on information needs were extracted and an analytic induction process using key words and statements was used to define needs for performance intelligence.

To answer research question 2, pieces of transcript were extracted that relate to the data organizations collect, what exchanges of data are taking place and what information is used for governance. The extracts were categorized using an inductive approach. To get more insight in available cost data this analysis was supplemented with a feasibility analysis exploring available administrative data on purchased health and social care delivery for the purpose of governing an integrated care network. This feasibility analysis aimed to identify, using an available administrative data source, what performance intelligence on costs is feasible for the Amsterdam Noord district. First, using the interface of the database, the available indicators, metadata, source information, and possible breakdowns were studied to assess what performance intelligence is currently available for the municipality of Amsterdam. Then via a series of meetings with Vektis a feasibility analysis for the Amsterdam Noord district was constructed from the database focusing on health and social service consumption. In the analysis specific attention was given to what level of data (neighbourhood, quarter or district) is informative to govern integrated care and the (im) possibilities of combining data from different sources (e.g. Social Support Act data with Healthcare insurance Act data), breakdowns (e.g. age groups), and benchmarks (e.g. Amsterdam Noord district compared to the municipality of Amsterdam excluding the Noord district).

Finally, to answer research question 3, study findings were shared with the governing body of the KMA and a reflection meeting was organized to define priority domains for improving performance intelligence to govern the integrated care network. Notes were inducted to key messages from the discussion by one researcher and further complemented by the two researchers present in the reflection meeting.

## Results

### The Krijtmolen Alliantie and their stakeholders: performance intelligence needs for the integrated care network in the Amsterdam Noord district

At the time of this study the Krijtmolen Alliantie had 13 member organizations representing health and social care provision organizations active in the Amsterdam Noord district. Selected stakeholders for interviews (*n* = 12) include the health insurer Zilveren Kruis, and the municipality of Amsterdam (both funders in health and social care in the Amsterdam Noord district), patient and client representation, the Dutch Healthcare Authority (NZa), general practitioners, and the ambulant pharmacy of the local hospital (see Additional file 1 for details on interviewees and catchment areas). KMA members reported working with a variety of catchment areas ranging from provision at neighbourhood level within the district, to province level. The KMA members and stakeholders serve different proportions of the population of Amsterdam Noord either due to the nature of their provision (for example: welfare aid to those in financial problems) and/or due to sharing the market in the district (for example with competing elderly care providers Amstelring, Cordaan and Evean). Multiple KMA members report overlapping service provision, stating potential for competition, as well as beneficial alignment, between providers. KMA members and stakeholders showed aligned needs for performance intelligence. The most commonly expressed needs for performance intelligence among interviewees were related to: 1) population data and outcomes adjusted to their catchment area, 2) information on the alignment between providers, 3) outcomes of (multiple-provider) interventions, and 4) an overview of health and social care information of and for the citizen/patient.

#### Population data and outcomes adjusted to catchment areas

Most health and social care providers report a need for information about the population they serve, either to be able to better define and support their target group, or as an outcome indicator for their work. Only few referred to the availability of population data in the Amsterdam Noord region, and the ones that did, found it hard to align the information with their practice as it is presented on an aggregated level and not tailored to their catchment areas.

#### Information on the alignment between providers

Most interviewees reported a lack of information on the alignment between providers. KMA members report that they are not always aware if other providers are involved with their patient/client. Most interviewees mention there is a barrier between social care information and health care information either on micro (individual patient), meso (between organizations) or macro (alignment social and health budgets) level. Some interviewees emphasized the importance of the correlation between the two, stating that coordinating health and social care in an integrated way can change decisions for individual treatment and adjust service provision on a meso and macro level.

#### Information on outcomes of (multiple-provider) interventions

Interviewees report a need for insights into the efficacy and efficiency of implemented multi-provider interventions. This performance intelligence can generate a learning curve on these interventions (mostly stated by providers of care) or can be used for accountability purposes (mostly stated by financers and patient representatives).

#### Overview of health and social care information for the citizen/patient

The interviewees representing patients in the region reported a need for an overview of health and social care data. They describe having limited information on possible health and social care providers in their region as well as lacking an overview of their own health and social care usage. Available information is compartmentalized per provider in, among other things, bilateral digital patient portals and limited in content (e.g. summarized letters or appointment dates). The interviewees representing patients in the region report that their main source for health and social care information comes from direct contact with their doctor, general practitioner (GP) or specialist, and experiences of friends and family.

### Available data (infrastructure) on health and social care in the Amsterdam Noord district and existing exchanges of data

#### Data collected in health and social care organizations

Interviewees describe data collection within their organizations that can be categorized as being used for five different purposes: 1) Identification and contact details of clients (name, address, personal identifier); 2) Treatment support (what are the needs of the client, what treatment is given). Every provider makes their own comprehensive analysis of the client, even after referral from other health and social care providers. Reasons mentioned to do this were incomplete information from referral in order to start health or social care provision, trust in referral information, and to encompass changes over time that might have occurred; 3) General management information for the organization, e.g. wellbeing of employees (e.g. absence due to illness of employees), quality (e.g. near misses, complaints), service (e.g. time until phone is being answered), production (e.g. amount of treatments done or clients seen), and finances (e.g. costs and benefits); 4) Financial accountability (different per stakeholder varying from minute writing of professionals to complex coded accounting); and 5) Quality of care outcome measurements (e.g. routine outcome measurements, patient reported outcome measures). When done, providers collect their own outcome measures, aimed at particular interventions within the institution and its target population. Many state to obtain most information, even in the case of a referral, from the patient themselves.

#### Data exchange between health and social care organizations

All health and social care providers exchange information mostly on an aggregated (organizational) meso-level with their funders and their relevant overseeing authorities (e.g. the Dutch Health Authority and/or Inspectorate of Health and Youth (IGJ)) in line with Dutch legislation. Structural exchange of information between health and social care providers (e.g. including patient identifier) is limited. The general practitioners, functioning as gate keepers, exchange most information with other providers via referrals (e.g. the referral from general practitioners to medical specialists through a referral data exchange programme (Zorgdomein)). However, general practitioners state an incomplete exchange of information with elderly care providers and social care providers, noting this is not standardized and only available circumstantially or on the initiative of certain care professionals. Health insurance companies have the possibility to integrate data on patient-level from different providers, however strictly monitored by privacy laws, and only for the percentage of patients that they finance (their market share). A couple of providers of those interviewed have agreements to exchange anonymized aggregated data with academic networks to contribute to national-level data for performance measurement of the overall health system, for example exchanges with the Academic Collaborative Centre for Elderly Care, the Academic Collaborative Centre for General Practice and the Netherlands Institute for Health Services Research (NIVEL). There is potential to exchange health and social care data on citizen level as most providers use personal identifiers in their registration.

The patient is the only point where all data for an individual comes together, however, this information is fragmented in bilateral communication between patient and providers and often summarized in referral letters or discharge letters formats. Patients can choose between one of the, at the time of this study, 29 accredited providers which all offer a ‘personal health environment’ (persoonlijke gezondheidsomgeving; PGO), to gather all of their individual healthcare data. Furthermore, there is a national healthcare data exchange platform (Landelijk Schakelpunt; LSP). This platform gives an overview to the patient what data (for example medication list) has been requested by providers. This platform, however, does not contain content data on care provided. Most health and social care providers report the existence of a patient portal or information exchange platform to communicate and/or share data bilaterally. All health and social care providers report internal data collection supporting their care provision. As providers do their own purchasing for digital support software, there is a great variety in digital support software and suppliers used by health and social care providers. An example being that there are eight different systems used by general practitioners in the district.

### Available performance intelligence to support triple aim goals

#### On population health

The descriptive data in the ‘Gemeentezorgspiegel’ and public data from the municipality of Amsterdam and the Public Health Service of Amsterdam (GGD) include more than 100 indicators on population health and welfare available about the Amsterdam Noord district. Only few of the interviewed KMA members mentioned this information and the ones that did, expressed to experience difficulties to use this information. Only one social care provider mentioned the use of neighbourhood-level data on income from the publicly available data combined with their own data gives them actionable insights. Multiple interviewees refer to the reports made by independent research institutes or the municipality. These reports give insights, but there remains a gap towards actionability, not aligning to the catchment areas or information needs of providers.

#### On costs per capita

Financial and usage data on health and social care are siloed and costs per patient or inhabitant over the whole spectrum of care are not integrated on patient identifier level due to privacy concerns, reducing the value for use. Costs for long-term care (Wet Langdurige Zorg; WLZ), medical care (Zorgverzekeringswet; Zvw) and social support (Wet Maatschappelijke ondersteuning; WMO) can be shown separately with breakdowns (e.g. age groups) and benchmarks (e.g. municipality vs national). The absence of integration of costs on personal identifier level makes it difficult to construct one indicator on per capita costs for governing integrated care delivery purposes.

Using either cost per inhabitant or cost per patient as an indicator for costs in the district, measure different things and show different outcomes. In the feasibility analysis of the Health Insurance Act claims, which includes the reimbursement of hospital care, GP’s, pharmaceuticals and mental healthcare the benchmark between Amsterdam Noord and Amsterdam excluding the Noord district, showed relative less costs per patient, but more cost per inhabitant. At the time of the study there is still a lag of about 1 year in reporting based on this database. To support governance of integrated care networks, information will have to be timelier.

#### On care experiences

Experience of care is documented in bilateral relations between KMA members and patients through patient satisfaction surveys, requests to (anonymously) review care givers or organizations and compliments and complaints reporting systems. There is a national system for reviewing healthcare providers called ‘Zorgkaart Nederland’ [25], however this system also measures patient experience per siloed provider (organization or specific professional). There is no data collection that gives insight in integrated patient experience in the Amsterdam Noord district.

### Governing an integrated health and social care district – priority domains for improvement of performance intelligence

In the reflection meeting with the board of the KMA, 7 members were represented. Reflecting on the findings, the fragmented data collection mirrors the fragmented accountability and financing siloes currently applied in the Netherlands. As one member put it: “die fragmentatie, ook in verantwoording, nekt ons”, loosely translated: “this fragmentation, also in accountability, is our downfall”. Governing to incentivize the Triple Aim in an integrated health and social care provision network requires an integrated data infrastructure that aligns with the governing structure. Board members emphasise that there is a willingness to integrate care provision, however this must be aligned with external expectations in accountability and financing. Board members reflect that to counteract the individual accountability and financing siloes, boldness is needed to step away from the norms and set up a performance intelligence dashboard that reflects the governing ambition. Multi-year contracts of involved parties are needed to create such movement space to innovate. Making the best use of available data is the first step towards building performance intelligence. The areas that were highlighted in the reflection meeting as potential domains for improving performance intelligence to govern the integrated care network were: costs, effectiveness & safety, patient experiences & outcomes, usage and process of care provision, cooperation & capacity in the network, evaluation of interventions, and population outcomes. There were some important reflections on these domains. The analytical capacity needed to process data into pieces of information and performance intelligence for a person centred approach, outreaches the capacity of one organization. The fragmentation in bilateral patient portals and patient experience measures per organization does not reflect a person centred approach. The current Dutch funding system is making use of budgets per organization, which does not allow for changing capacity needs within an integrated care network to carry over funding from one organization to the other. The timeliness of Dutch administrative reporting systems for care, needs to improve in order to create indicators that are actionable for governance. The reflection meeting defined costs (combined accountability through aligning financial incentives) and patient experiences & outcomes (measured in an integrated approach) as priority domains to improve the performance intelligence of their integrated care network.

## Discussion

Our findings show that the performance intelligence in order to govern regional integrated provision of health and social care in the Netherlands is still showing considerable barriers on both data availability and translating data into actionable indicators for governance. It is known that integrated care can contribute to a more people centred approach [26]. Our findings suggest that intentions for integrating health and social care services are there, but the data and information structure needed to govern such a network are not. The identified barriers in data availability and data alignment for effective governance in the integrated care network in our case study can partially be explained by the competitive market structure introduced in healthcare in the Netherlands in 2006 and 2008 [27]. Looking at the information needs, data currently collected and shared, and priority domains to improve performance intelligence of the Amsterdam Noord alliance we found that alignment with external expectations in financing and the current Dutch funding system of budgets per organization does not allow to carry over funding from one organization to the other when capacity needs within an integrated care network change. A study by De Vries et al. [28] studied barriers to payment reforms in nine population management sites in the Netherlands. They found that information asymmetry, perceived by both the financers and the providers as being in favour of the other, was an important barrier for financial reform towards population management strategies. Our findings show that financers have an information need for outcomes of integrated care provision and that the Dutch administrative data does not allow for an integrated cost per capita indicator. Reflecting on these findings, breaking the chicken and egg cycle between willingness to structurally finance integrated care initiatives and meeting information needs might be an important strategy to align stakeholders in integrated networks. Pilot initiatives where different funding streams are pooled and agreements are reached between the network of providers with the financiers (insurers, municipality) on performance measures based accountability, can be the way forward. For Amsterdam Noord these discussions on alternative financing arrangements are taking place.

There are an increasing number of international examples of similar attempt to integrate care. However, expanding in the direction of social care and upscaling local initiatives is still unusual as many challenges exist due to misalignment in culture, governance, and information infrastructure [29, 30]. International evidence shows that the use of feedback information and a shared savings model like in the Healthy Kinzigtal region in Germany [31], cultural integration in local initiatives in Finland [32] and New Zealand [33] and multi-level policy interventions in the Basque region [34, 35] are long-term investments that can boost performance of integrated care networks. The strengths of a network like the Krijtmolen Alliantie is their 10 year investment in mutual trust and vision between health and social care providers to build the foundation of its integrating care network. This investment creates a strong base to build towards a bottom-up person centred, population based model of care for the Netherlands. As health systems worldwide are changing to more cooperative integrated networks, data infrastructures should align to support governance in networks with actionable performance intelligence. The importance of an integrated data infrastructure, feeding agreed upon performance intelligence for decision making has become even more clear in the light of the COVID-19 pandemic [9]. Integrated national data infrastructures like the Danish national patient registry have been able to inform decision makers on the effect of the pandemic on non-COVID-19 related care needs, and thus were able to make a more informed decision on COVID-19 measures from an integrated perspective [36]. Our recommendation is to align performance intelligence with the regionalized responsibilities for governance of health and social care.

Our findings show that care providers may have become reluctant to share essential data for integrating health and social care treatment, hindering the development of an integrated data and knowledge infrastructure, essential to govern effectively across organizational silos. We acknowledge the importance of protecting sensitive health and social care data. The bottleneck for governance does not seem to be with having to identify the specific patient, but the need to integrate data on patient level to gain actionable insights. This leaves room to organize data for effective regional governance within the scope of the General Data Protection Regulation (GDPR) taking into account the privacy of the patient [37].

### Strengths and limitations

KMA members were well represented in the study by interviews and the reflection meeting, and their most important stakeholders were able to contribute. At this moment youth care and independent pharmacies are not part of the KMA alliance, and therefore not taken into account in this study. This study is done in the Dutch context, however can be an inspiration to other high income countries moving from a competitive healthcare market towards regional governance of integrated care.

## Conclusion

As health systems are changing to more cooperative integrated networks, data infrastructures should align in order to support governance on performance intelligence. Available information lacks actionability for the governance of integrated care networks. In this case study an information need was observed in the following areas: 1) population data and outcomes adjusted to catchment areas of providers, 2) data on alignment between providers, 3) outcomes of (multiple-provider) interventions, and 4) an overview of health and social care information of and for the citizen/patient. There are indicators on population health and welfare, however they have limited actionability for providers due to a misalignment with their respective catchment areas. Indicators to measure integrated cost per capita and per patient, and integrated patient experience/outcomes indicators are lacking. Priority domains to improve the performance intelligence of the integrated care network proposed by this case study are: 1) Per capita and per patient cost data integration that would allow combined accountability through aligning financial incentives to facilitate integrated care of financing, and 2) combined patient experience and outcome measures to reflect network quality of care and patient experience performance. Multiple-year contractual arrangements with funding agencies, integrated regional data performance infrastructure, and formalized regional patient and citizen representation could facilitate the further development of integrated health and social care delivery networks in the Netherlands.

## Supplementary Information

**Additional file 1.** Validated interviewee list with catchment areas of organizations.

**Additional file 2.** Translated interview guide.

## Data Availability

The data that support the findings of this study are stored at the AMR research B.V. and available for quality inspection. However restrictions apply to the public availability of this data. Interview transcriptions and reflection meeting notes cannot be anonymized due to the thick content of the material. Anonymized data used for the feasibility analysis are available from the authors upon reasonable request and with permission of the KMA, Vektis and the municipality of Amsterdam.

## Abbreviations

GGD: Gemeentelijke Gezondheidsdienst
GP: General Practitioner
IGJ: Inspectie voor Gezondheidszorg en Jeugd
KMA: Krijtmolen Alliantie
LSP: Landelijk Schakelpunt
NZa: Nederlandse Zorgautoriteit
PGO: Persoonlijke Gezondheidsomgeving
WLZ: Wet Langdurige zorg
WMO: Wet Maatschappelijke ondersteuning
ZVW: Zorgverzekeringswet
