# Supplementary material for: Improving performance intelligence for governing an integrated health and social care delivery network: a case study on the Amsterdam Noord district
Source: BMC Health Serv Res. 2021 May 28;21:517. doi: 10.1186/s12913-021-06558-2 (PMC8160080; doi:10.1186/s12913-021-06558-2)
Supplement: Supplementary file 1 — Additional file 1. Validated interviewee list with catchment areas of organizations. [file 12913_2021_6558_MOESM1_ESM.docx]

# Improving performance intelligence for governing an integrated health and social care delivery network: a case study on the Amsterdam Noord district

## Véronique LLC Bos*, Niek S Klazinga* and Dionne S Kringos*

*Amsterdam UMC, University of Amsterdam, Department of Public and Occupational Health, Amsterdam Public Health research institute, Amsterdam, the Netherlands.

## Appendix 1 validated interviewee list with catchment areas of organizations

For the privacy of the interviewees the interviewee list is shown at organizational level, not at individual interviewee level. When interviewed medical specialists were affiliated to an organization they are not shown separately from the interviewed CEO’s, even though two separate interviews were conducted.

| KMA member organisations | Type of organisation | Catchment area |
| --- | --- | --- |
| Amstelring | long term care provider, elderly care provider | Primarily Amsterdam, Haarlemmermeer, Amstelveen, Nieuw-Vennep, Uithoorn and Diemen, and to a smaller extent Amsterdam Noord. |
| Arkin | mental health care provider | Primarily Amsterdam, Amersfoort en Utrecht. About 1.500 à 2.000 clients per year in Amsterdam Noord. |
| BovenIJ hospital | hospital care provider | Primarily Amsterdam Noord and neighbouring areas. |
| Cordaan | long term care provider, elderly care provider, mental health care provider, and provider of care for persons with disabilities | Primarily Amsterdam, Huizen, Diemen en Nieuw-Vennep. Relatively big provider in Amsterdam Noord (ambulant care and two elderly care facilities). |
| Doras | social work provider | Primarily Amsterdam Noord and since 2016 also Amsterdam Zuid (merger with Puur Zuid). |
| Evean | long term care provider, elderly care provider | Primarily Noord-Holland province, four locations in in Amsterdam Noord. |
| HVO Querido | social care provider | Primarily Amsterdam and Haarlem. Relatively small provider in Amsterdam Noord district. |
| Stichting Amsterdamse Gezondheidscentra | ambulant health care centre | Primarily Amsterdam (16 centres), and to a smaller extend Amsterdam Noord (1 centre, serving approx. 6.000 inhabitants). |

| Stakeholders | Type of organisation | Catchment area |
| --- | --- | --- |
| Cliëntenbelang Amsterdam | patient representation | All Amsterdam inhabitants with a psychiatric, physical or mental disorder, the chronic ill, vulnerable elderly and informal care givers. |
| The Client Advisory Board of the BovenIJ hospital | patient representation | Representating all patients of the BovenIJ hospital |
| FysioHolland | paramedical health care provider | FysioHolland is the biggest Physiotherapy provider in the Netherlands, with multiple locations in Amsterdam Noord. |
| Municipality of Amsterdam | municipality and social and long term care financer | Serves municipality of Amsterdam |
| Public Health Service (GGD) of Amsterdam | municipal public health services | Includes the municipalities Aalsmeer, Amsterdam, Ouder-Amstel, Amstelveen, Diemen en Uithoorn |
| General practitioner | general practitioners | General practitioners in Amsterdam Noord, approx. serving about 2300 patients per practitioner. |
| MEE | independent client support, support for children with disabilities and their parents | In 2019 approx. 190 new clients in Amsterdam Noord. |
| Dutch Healthcare Authority (NZa) | Dutch Healthcare Authority | Representing all citizens nationwide. |
| Oupatient pharmacy BovenIJ hospital | ambulatory pharmacy situated in the hospital | Every person with a medication need in Amsterdam Noord, Oostzaan, Zaandam, Wormerveer & Landsmeer. |
| Zilveren Kruis | healthcare insurance agency | Operating on a national level, has aprox. 60% marketshare in Amsterdam. |
