# Supplementary material for: Improving performance intelligence for governing an integrated health and social care delivery network: a case study on the Amsterdam Noord district
Source: BMC Health Serv Res. 2021 May 28;21:517. doi: 10.1186/s12913-021-06558-2 (PMC8160080; doi:10.1186/s12913-021-06558-2)
Supplement: Supplementary file 2 — Additional file 2. Translated interview guide. [file 12913_2021_6558_MOESM2_ESM.docx]

# Improving performance intelligence for governing an integrated health and social care delivery network: a case study on the Amsterdam Noord district

## Véronique LLC Bos*, Niek S Klazinga* and Dionne S Kringos*

*Amsterdam UMC, University of Amsterdam, Department of Public and Occupational Health, Amsterdam Public Health research institute, Amsterdam, the Netherlands.

## Appendix 2 – translated interview guide

START

Thank you for your interest in this research

Give an explanation of the research

Read through informed consent (provided it has not been returned signed yet)

Start audio recording

• What is your organization and what role do you have in Amsterdam Noord?

• What is the population you serve?

• Which (healthcare) data / information do you register?

• With whom do you share (healthcare) data / information?

• Which performance indicators / management information do you use in your organization?

• Are you involved in initiatives for the exchange of data in the Amsterdam North region to increase the quality of care, improve public health or reduce healthcare costs?

• What (healthcare) data / information / performance indicators do you need, or lack now, in order to better manage the quality of healthcare, public health and / or healthcare costs in the region?

• Do you experience limiting factors in obtaining (care) data / information / performance indicators to manage the quality of care, public health and / or healthcare costs in the region? What is needed to reduce these barriers?

• What results can be achieved by the missing (if previously mentioned) data, information or performance indicators? What do you hope to achieve by focusing on integrated data / information / performance indicators?

These were my questions, do you wish to share any additional information?

Thank you again for participating in the study and offering the opportunity to be kept informed about the results of the study.

Do you have any questions or comments about this interview or the research in general?

END
